# Cell-penetrating peptide-conjugated, splice-switching oligonucleotides mitigate the phenotype in *BTK*/*Tec* double deficient X-linked agammaglobulinemia model†

**DOI:** 10.1039/d4cb00312h

**Published:** 2025-03-31

**Authors:** Burcu Bestas, H. Yesid Estupiñán, Qing Wang, Shabnam Kharazi, Chenfei He, Dara K. Mohammad, Dhanu Gupta, Oscar P. B. Wiklander, Taavi Lehto, Karin E. Lundin, Anna Berglöf, Mikael C. I. Karlsson, Frank Abendroth, Samir El Andaloussi, Michael J. Gait, Matthew J. A. Wood, Christian J. Leumann, Dmitry A. Stetsenko, Robert Månsson, Jesper Wengel, Rula Zain, C. I. Edvard Smith

**Affiliations:** a Department of Laboratory Medicine, Karolinska Institutet, ANA Futura, Alfred Nobels Allé 8 Floor 8 SE-141 52 Huddinge Sweden edvard.smith@ki.se; b Department of Microbiology, Tumor and Cell Biology, Karolinska Institutet Stockholm Sweden; c Institute of Techology, University of Tartu, Tartu Estonia; d Medical Research Council Laboratory of Molecular Biology Cambridge CB2 0QH UK; e Karolinska ATMP Center, Karolinska Institutet, Karolinska University Hospital SE-171 76 Stockholm Sweden; f Department of Paediatrics, University of Oxford Oxford OX3 7TY UK; g Department of Chemistry and Biochemistry, University of Bern, Freiestrasse 3 CH-3012 Bern Switzerland; h Department of Clinical Immunology and Transfusion Medicine, Karolinska University Hospital Stockholm Sweden; i Institute of Cytology and Genetics, Siberian Branch of the Russian Academy of Sciences, 10 Lavrentiev Ave. Novosibirsk 630090 Russia; j Department of Physics, Novosibirsk State University, 2 Pirogov Str. Novosibirsk 630090 Russia; k Department of Physics, Chemistry and Pharmacy, Biomolecular Nanoscale Engineering Center, University of Southern Denmark Odense Denmark; l Centre for Rare Diseases, Department of Clinical Genetics and Genomics, Karolinska University Hospital SE-171 76 Stockholm Sweden; m Departamento de Ciencias Básicas, Universidad Industrial de Santander Bucaramanga Colombia; n Breast Center, Karolinska Comprehensive Cancer Center, Karolinska University Hospital Stockholm Sweden; o Department of Chemistry, University of Marburg Marburg D-35043 Germany; p Institute of Pharmacy, Faculty of Medicine, University of Tartu, Nooruse 1, 50411 Tartu Estonia

## Abstract

Splice-switching oligonucleotides (SSOs) have been developed as a treatment for various disorders, including Duchenne muscular dystrophy and spinal muscular atrophy. Here, the activity of several different SSOs was investigated as potential treatments for B lymphocyte disorders with a focus on X-linked agammaglobulinemia (XLA), caused by defects in the gene encoding Bruton's tyrosine kinase (*BTK*). In this study, the activity of locked nucleic acid (LNA), tricyclo-DNA (tcDNA), phosphoryl guanidine oligonucleotides (PGO) and phosphorodiamidate morpholino oligomers (PMO) were compared, targeting the pseudoexon region of *BTK* pre-mRNA. We further investigated the effect of conjugating cell-penetrating peptides, including Pip6a, to the SSOs. The effect was measured as splice-switching *in vitro* as well as in a further developed, bacterial artificial chromosome transgenic mouse model of XLA. Therapy in the form of intravenous infusions 2 times a week during 3 weeks of PMO oligomers conjugated to Pip6a was sufficient to partly restore the *in vivo* B lineage phenotype. SSOs treatment also provides a unique opportunity to get insights into a restoration process, when B lymphocytes of different maturation stages are simultaneously splice-corrected.

## Abstract 2

Splice-switching oligonucleotides (SSOs) targeting *BTK* pre-mRNA offer a potential therapy for X-linked agammaglobulinemia (XLA). PMO-Pip6a conjugate treatment partially restores the B lineage phenotype, offering insights into splice correction in B cell maturation.

## Graphical abstract

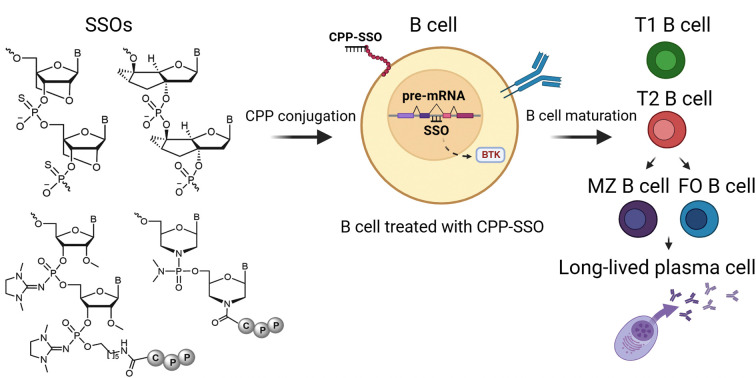

## Introduction

Splice-switching oligonucleotides (SSOs) interfering with pre-mRNA processing, first developed in 1993,^1^ have increasingly impacted the treatment of a number of genetic diseases. Consequently, SSOs have been approved as therapy in Duchenne muscular dystrophy,^2,3^ and such a strategy was recently used for the innovative treatment of a newly discovered neurodegenerative disorder, with only a single known, affected patient.^4,5^ This demonstrates the versatility and short time from the identification of a mutation to the actual development of a novel treatment modality. Furthermore, the perhaps most successful treatment to date is in spinal muscular atrophy. By administrating an SSO-based drug, spinraza, into the cerebrospinal fluid, repression of a splice silencer in the survival motor neuron gene 2 (*SMN2*) leads to the inclusion of exon 7 into the pre-mRNA with great clinical benefit.^6^

As with other forms of oligonucleotide (ON) therapies, the challenge is to successfully transfer sufficient numbers of SSOs into relevant cells in the disease-affected tissue. Hepatocytes and cells in the central nervous system can be readily targeted, but for many other organs uptake remains a hurdle.^7^ To this end, we have previously developed an experimental treatment strategy for X-linked agammaglobulinemia (XLA).^8^ In this B-lymphocyte disorder, a developmental block occurs at the pro-B to pre-B transition^9^ owing to that the *BTK* cytoplasmic protein-tyrosine kinase is lacking or functionally disabled.^10^ The blocked maturation results in an increased susceptibility to bacterial and enteroviral infections.^10,11^ By generating a *BTK*-transgenic mouse, which carries a mutated human bacterial artificial chromosome (BAC), and, simultaneously lacks the endogenous *BTK* kinase, it was possible to obtain an XLA mouse model to study the effect of SSOs.^8^ We observed that when targeting B-lineage cells, only SSOs carrying a cell-penetrating peptide were efficient for *in vivo* correction. In these studies, *BTK* protein synthesis was restored through the removal of a pseudo-exon erroneously introduced by a single point mutation. However, while our previous work demonstrated protein restoration, it did not assess the phenotypic and functional consequences of this correction *in vivo.*

We here analyze a set of chemistries for efficient pseudo-exon removal in B-lymphocytes and demonstrate for the first time that by repeated administration of cell-penetrating peptide SSO conjugates, it is possible to partly restore the phenotype of the affected lymphocytes *in vivo*. Since *BTK* deficiency alone only results in a mild B-lineage defect in mice,^12–14^ we have used a *BTK*/*Tec* double knockout mouse strain lacking both of these kinases. This animal model manifests a more severe phenotype resembling XLA.^15^ Prior to treatment, spleen and lymph nodes of *Tec*/*BTK* double-deficient mice displayed a dramatic reduction of B cells compared with *BTK* single knockout mice. Similar to human settings, a relative decrease of the mature IgM^lo^IgD^hi^ population and accumulation of CD43^+^ (Pro-B) B cell precursors were noted, indicating a partial developmental block at the large pre-B cell stage.^15^ Thus, this study goes beyond protein restoration to assess phenotypic recovery in a *BTK*/*Tec* double knockout model, which more closely mimics human XLA, allowing us to evaluate functional B cell development after sustained SSO treatment.

Disorders affecting B lineage cells comprise a large part of human disease including autoimmunity, autoinflammation, allergies, leukemias&lymphomas, primary immunodeficiencies and infectious disease. Our study not only confirms the feasibility of targeting pseudoexon 4a with SSO-CPP conjugates but also provides the first *in vivo* evidence of functional restoration in a severe B-cell deficiency model. These findings broaden the applicability of ON-based therapeutics for genetic immunodeficiencies, highlighting their potential to address complex hematopoietic disorders beyond XLA. Hence, apart from addressing inherited loss-of-function disorders affecting the B-cell lineage, these studies may also be of interest for the general development of ON therapeutic strategies targeting B-lymphocyte-derived leukemias and lymphomas.

## Results and discussion

### *In vitro* comparison of oligonucleotides with different chemistries in a reporter cell line

**Fig. 1:**
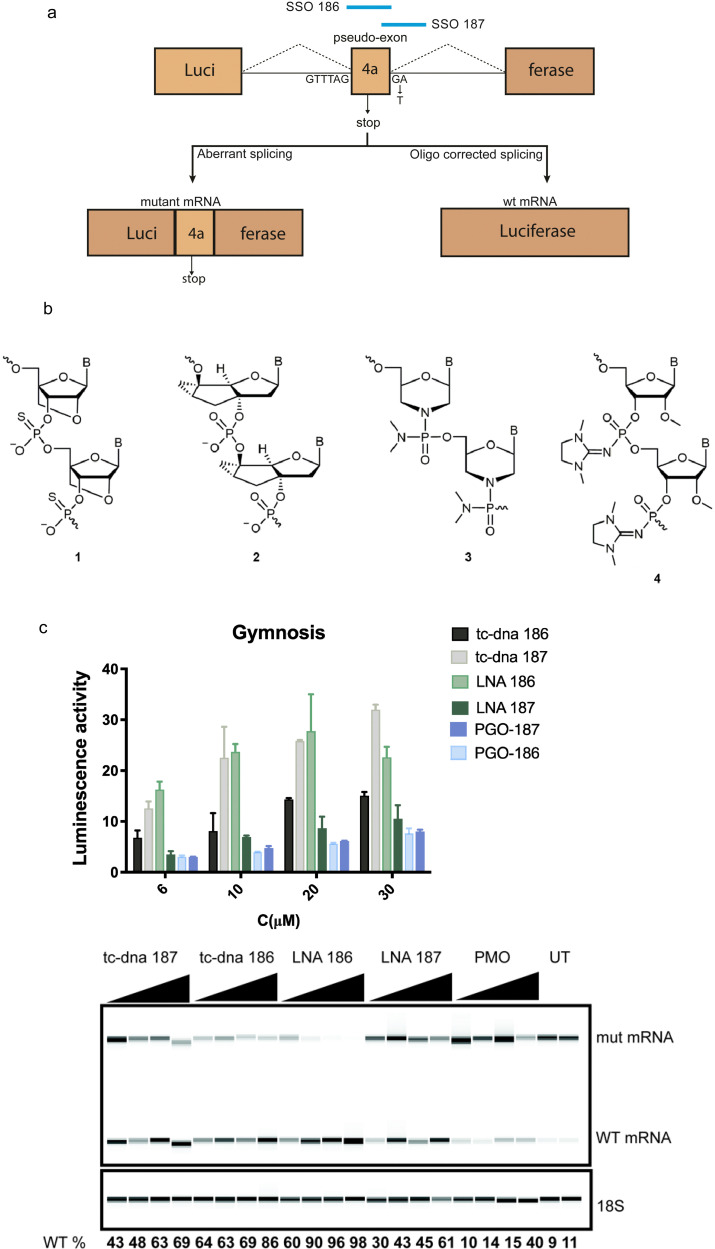
Gymnosis activity of SSOs with different chemistries in a reporter cell line model. (a) Outcome of predicted splicing in the reporter luciferase U2OS cell line model with a pseudo-exon-interrupted (exon 4a) mutated *BTK* intron 4, SSO treatment leads to restored splicing and generation of functional luciferase protein. (b) Structures of oligonucleotide analogues used in the study: (1) locked nucleic acid (LNA) phosphorothioate; (2) tricyclo-DNA (tcDNA) phosphodiester; (3) phosphorodiamidate morpholino oligomer (PMO); (4) phosphoryl guanidine oligo-2′-*O*-methylribonucleotide (2′-OMe PGO). (c) The upper panel shows the luciferase activity 24 h after SSO transfection. Tricyclo-DNA (tcDNA), locked nucleic acid (LNA) and PGO modified SSOs were tested. Data represent mean + SD of 2 independent experiments, each with 2 replicates. The lower panel shows a representative RT-PCR result of the same experiment. 389-bp and 271-bp bands correspond to aberrant (mut) and corrected (with *BTK* intron 4 excised) mRNA bands, respectively. The lower, 18S ribosomal RNA band served as control. The percentage of WT *BTK* RNA was calculated as WT RNA fraction × 100/(mis-spliced + WT RNA fractions). PMO = phosphorodiamidate morpholino oligomer and UT = untreated control. The corrected peak area was used for the calculation.

The strategy for pseudo-exon (exon 4a) exclusion is depicted in Fig. 1a. We previously screened and optimized a large number of SSOs based on locked nucleic acid (LNA) and phosphorodiamidate morpholino oligomer (PMO) chemistries spanning the intron 4 of the human *BTK* gene.^8^ The most efficient SSO, named 186, targeting the predicted exon splice enhancer (ESE) and another SSO targeting the 5′-splice site (named 187) were chosen for the current study. Owing to that our previous studies were restricted to LNA-DNA mixmers and PMOs,^8^ we here wanted to extend the analysis to other types of nucleotides. Different chemistries of both the 186- and the 187-series of SSOs were first tested in a U2OS reporter cell line carrying an interrupted luciferase gene with the mutated patient-derived *BTK* intron 4. The first sets of SSOs were synthesized with tricyclo-DNA (tcDNA) chemistry, which has been shown to have a potent activity in a dystrophic mouse model.^16^ A second set of SSOs was synthesized as novel phosphoryl guanidine oligonucleotides (PGO) as charge-neutral phosphate mimics. The PGOs represent a third class of charge-neutral oligonucleotide derivatives, alongside the previously known peptide nucleic acids (PNA) and morpholino oligomers (PMO).^17^ This chemistry has not been tested in the context of splice-switching in B lymphocytes up to date but for antisense properties including gapmers^18^ and as well as G-quadruplex-forming agents.^19^ The structures of these SSOs are shown in Fig. 1b. The SSOs were transfected into reporter cells *via* gymnosis^20^ and compared to the previously described LNA-modified SSOs, with the splice-switching activity calculated based on the luminescence as well as RT-PCR. Treatment of the reporter cells showed that the LNA-DNA mixmer SSO 186 and tcDNA modified SSO 187 display the highest activity (Fig. 1c). Although the unconjugated phosphoryl guanidine (PGO) SSOs demonstrated a dose response, the overall *in vitro* activity was much lower compared to other chemistries (Fig. 1c).

### CPP-mediated delivery of the SSOs in a reporter cell line

**Fig. 2:**
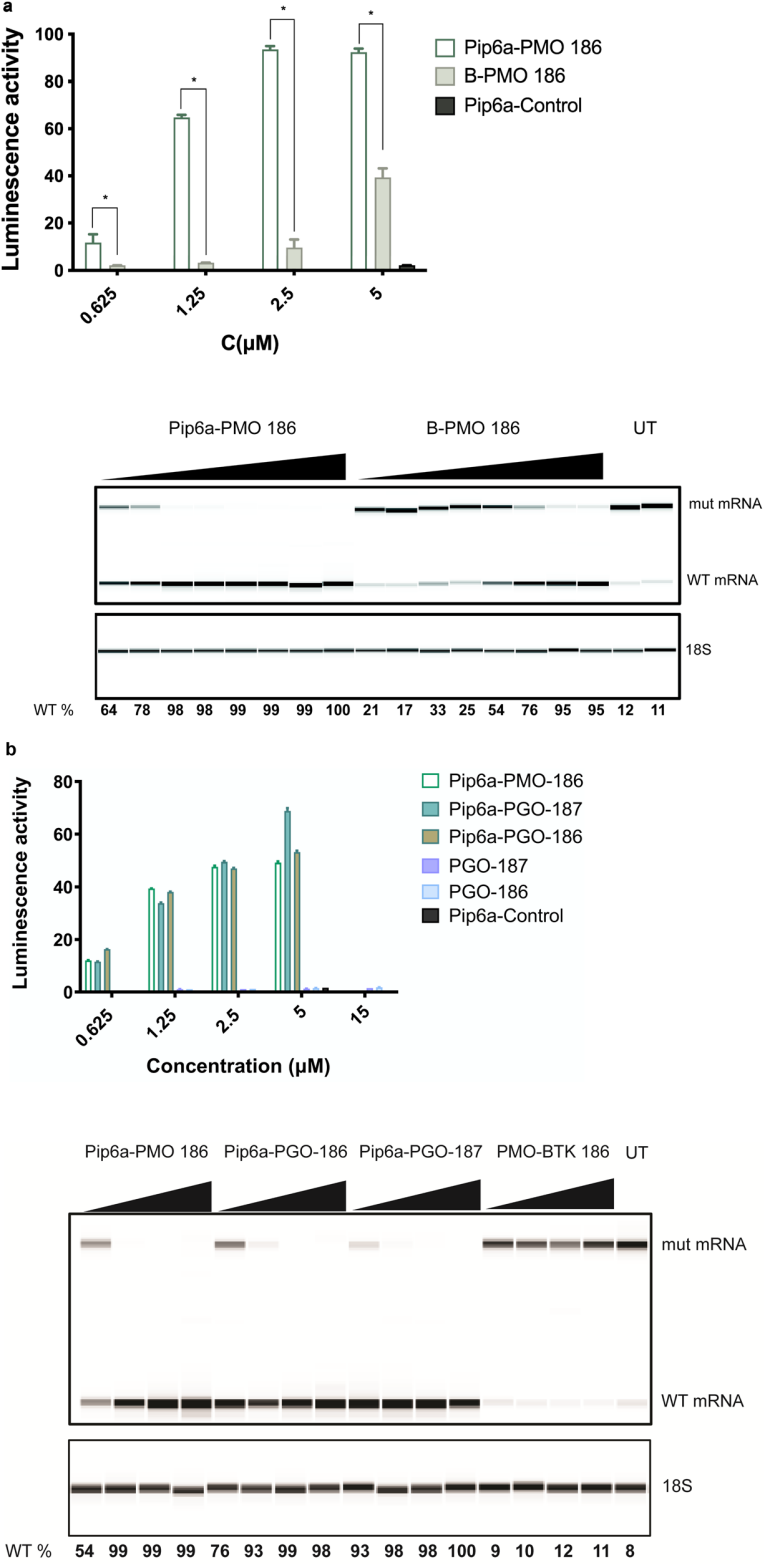
CPP-mediated delivery in a reporter cell line. (a) Comparison of Pip6a-PMO 186 with B-PMO 186 as measured by luciferase activity 24 h after transfection. (b) Increase in luciferase activity by Pip6a-PMO and by unconjugated and Pip6a-conjugated phosphoryl guanidine (PGO) SSOs. The lower panels (a) and (b) show RT-PCR results for aberrant and corrected mRNA. PMO-*BTK* is the unconjugated 186.25^8^ and Pip6a-Control is dystrophin targeting control oligonucleotide. Data represent mean + SD of 2 independent experiments, each with 2 replicates. A dosage of 15 μM was only used for PGO-186 and PGO-187 and a dosage of 5 μM was used for Pip6a-Control. 389-bp and 271-bp bands correspond to aberrant and corrected (with *BTK* intron 4 excised) mRNA bands, respectively. The 18S serves as an RNA quality control. The percentage of WT *BTK* RNA was calculated as WT RNA fraction × 100/(mis-spliced + WT RNA fractions). The corrected peak area was used for the calculation. Two-tailed Mann–Whitney test was used to calculate significance, *P ≤ 0.05.

We previously studied the effect of PMO-based ONs conjugated to a cell-penetrating peptide.^8^ In order to determine whether the cell-penetrating peptide (CPP) conjugates could further improve the splice-switching activity, we decided to test Pip6a-PMO, an improved derivative of Pip5e-PMO with alterations in the hydrophobic core, which has shown the ability to restore dystrophin function in *mdx* mouse model.^21,22^ A more recent study using Pip6a-PMO targeting the pathologic CAG repeats in myotonic dystrophy type 1 (DM1) mice showed normalization of the disease transcriptome and phenotype.^23^ To this end, we used the B-PMO 186 as the most efficient conjugate from our previous study^8^ and compared it to Pip6a-PMO 186. Pip6a was previously never tested for SSO transfer into B lymphocytes. The comparison between the two derivatives showed a striking difference as Pip6a-PMO 186 demonstrated superior activity over B-PMO 186, even at lower concentration ranges both on protein and RNA levels (Fig. 2a). This prompted us to also test the novel phosphoryl guanidine (PGO) modification with the same conjugation, which showed several fold increase in activity with the overall efficiency being similar to the Pip6a-PMO conjugates. Moreover, Pip6a-Control targeting the dystrophin locus as a control and PMO-*BTK* 186 without CPP conjugation did not show any effect (Fig. 2a and b). Due to the uncharged backbone of PMO and PGO SSOs, they were chosen to be conjugated to positively charged Pip6a cell-penetrating peptide.

### CPP-conjugated SSOs induce splice-correction *ex vivo* in primary B cells

**Fig. 3:**
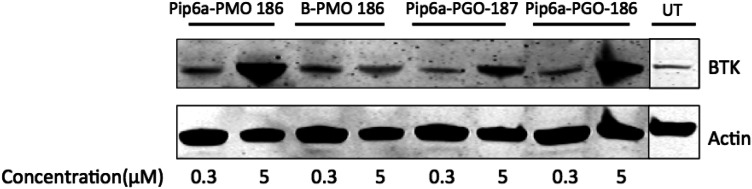
*Ex vivo* treatment of primary B cells with 0.3 μM and 5 μM of the splice-switching oligonucleotides (SSOs) Pip6a-PMO 186, B-PMO 186, Pip6a-PGO-187 and Pip6a-PGO-186 results in detectable *BTK* protein. Western blot shows a representative experiment of *BTK* protein after 48 h from 2 independent tests. Actin served as loading control.

In order to test the activity of the SSOs in a physiological, genomic setting, we first isolated primary B cells from mice expressing the mutant *BTK* transcript. We subsequently treated cells with the CPP-conjugated SSOs (Pip6a-PMO 186, B-PMO 186, Pip6a-PGO-187 and Pip6a-PGO-186) using two different doses (0.3 μM and 5 μM) and incubated cells for 48 h. Following the incubation, primary B cells were collected and *BTK* expression was evaluated by western blot. As shown in Fig. 3, the two Pip6a 186 conjugates with the different chemistries demonstrated the most efficient restoration of *BTK* protein.

### Pip6a-PMO restores *BTK in vivo* upon systemic delivery

The most efficient Pip6a-PMO 186 conjugate was subsequently injected intravenously into our previously described BAC transgenic mice,^8^ carrying the entire human mutant *BTK* locus in its germline and with an inactivated mouse *BTK* gene thereby preventing expression of endogenous mouse *BTK* protein.

**Fig. 4:**
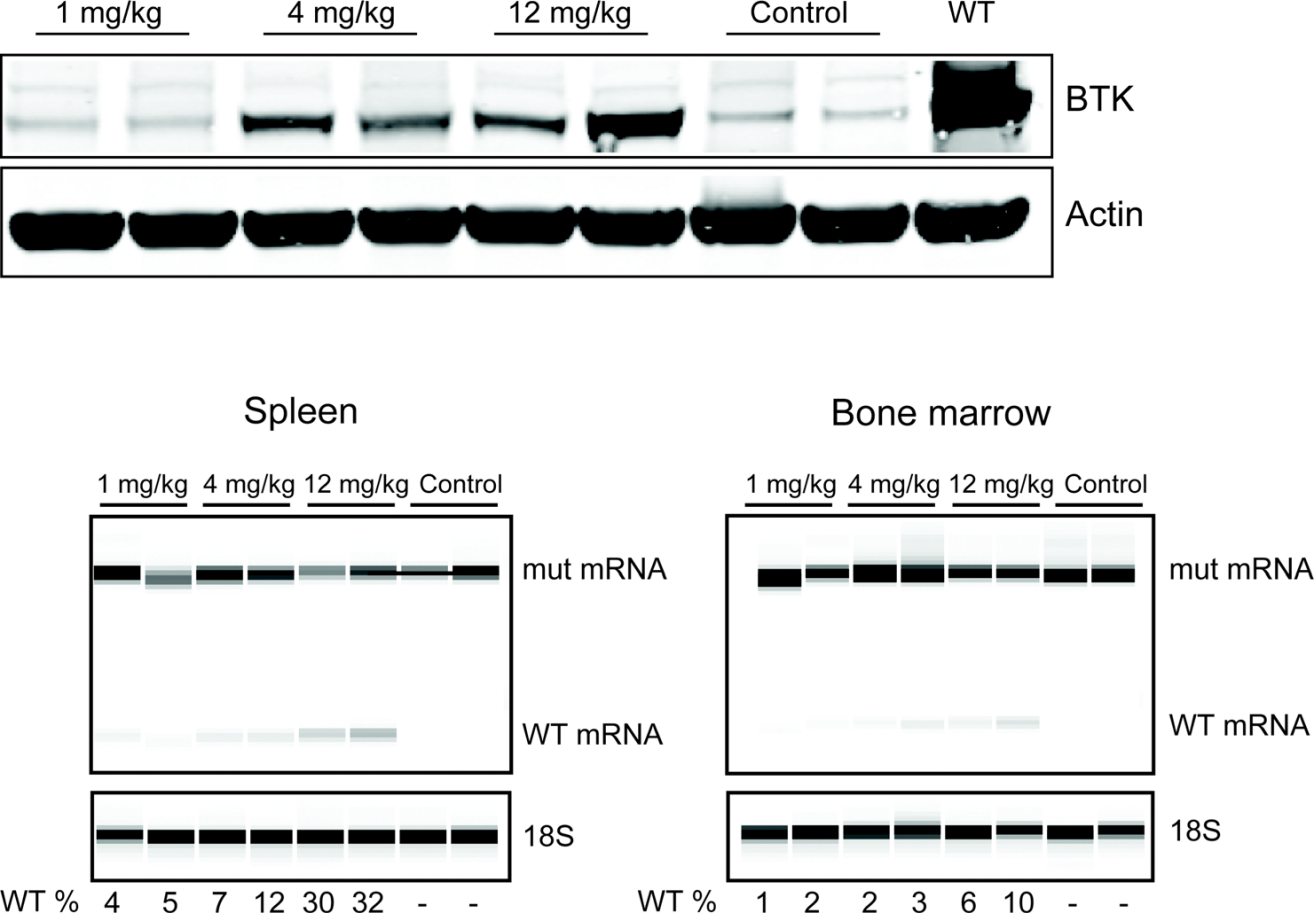
*In vivo*, injection of Pip-6a PMO 186 into the BAC transgenic mice restores the *BTK* expression. The upper panel shows the *BTK* expression in splenic B cells by western blot. The lower panel shows the restoration of the WT mRNA in B cells isolated from the spleen (left) and the bone marrow (right) of the transgenic mouse after the indicated treatments. The percentage of WT *BTK* RNA was calculated as WT RNA fraction × 100/(mis-spliced + WT RNA fractions). The corrected peak area was used for the calculation. Two mice were injected for each dose.

The correction of the mutant, human *BTK* mRNA in this mouse model generates a functional human *BTK* protein,^8^ which is almost identical to the mouse protein.^24^ In order to find the most optimal dose, different concentrations were intravenously injected and after 48 h, B cells from the spleen and the bone marrow of the mice were isolated. As depicted in Fig. 4, efficient *BTK* restoration was achieved already at 4 mg kg^−1^, which was also confirmed by the RT-PCR results (lower panel). This data overall suggests that Pip6a-PMO 186 is efficient already at lower doses. The biodistribution of Pip-series has been studied before;^25,26^ however, this is the first time it has been shown to be effective for the systemic treatment of B cells *in vivo*.

### Pip6a-PMOs rescue the phenotype in the *BTK*/*Tec* double knock-out, BAC-transgenic mouse model

In order to assess the functionality of the most efficient conjugate, we injected 10 mg kg^−1^ Pip6a-PMO 186 as intravenous infusions 2 times a week during 3 weeks into *BTK*/*Tec* double-deficient mice. Three weeks later, cells from the spleen and bone marrow as well as sections of the spleen were collected for FACS and immunohistochemistry analysis.

**Fig. 5:**
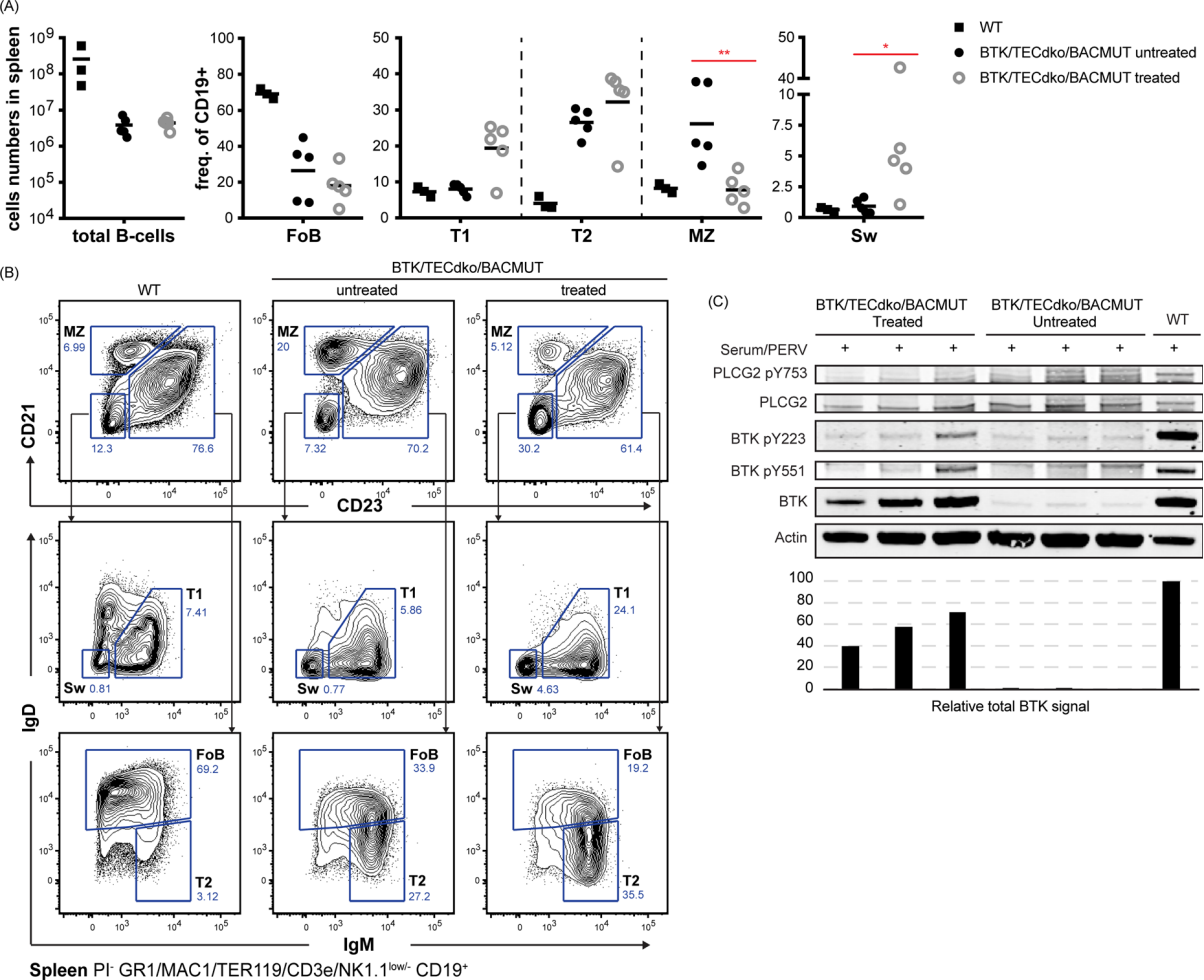
Characterization of the spleen B cell compartment following treatment with Pip6a-conjugated splice-switching PMOs. Panel (A), total (B) cells and B-cell subpopulations, Follicular (FoB), transitional type 1 (T1), transitional type 2 (T2) marginal zone (MZ), and switched (sw) cells. Panel B, Gating strategy for identification of indicated B-cell subsets. Prior gating was performed as indicated below the fluorescence-activated cell sorting plots. Propidium iodide (PI) was used for dead cell discrimination. Panel (C), western blot showing expression of total *BTK*, PLCG2 and actin (loading control) as well as activated forms as detected by phosphorylation-specific antibodies for *BTK* pY223 and pY551 and PLCG2 pY753. The total *BTK* levels are presented in the bar graph.

We observed significant changes in switched and marginal zone (MZ) B-cells in the spleen (Fig. 5A and B). Interestingly, the frequency of MZ B-cells was reduced in treated animals and the frequency of these cells was higher in the *BTK*/*Tec* double-deficient animals compared to WT. This is in accordance with previously published data on untreated mice^27^ and suggests normalization of the phenotype after therapy. We also observed significantly increased numbers of switched B-cells (Fig. 5A and B) and an increased tendency to develop a higher level of the T1 B-cell subset (Fig. 5A and B). To this end, Follicular (FoB) cells and the T2 B-cell subset did not show significant changes. *BTK* expression as measured by WB was confirmed in treated mice (C).

**Fig. 6:**
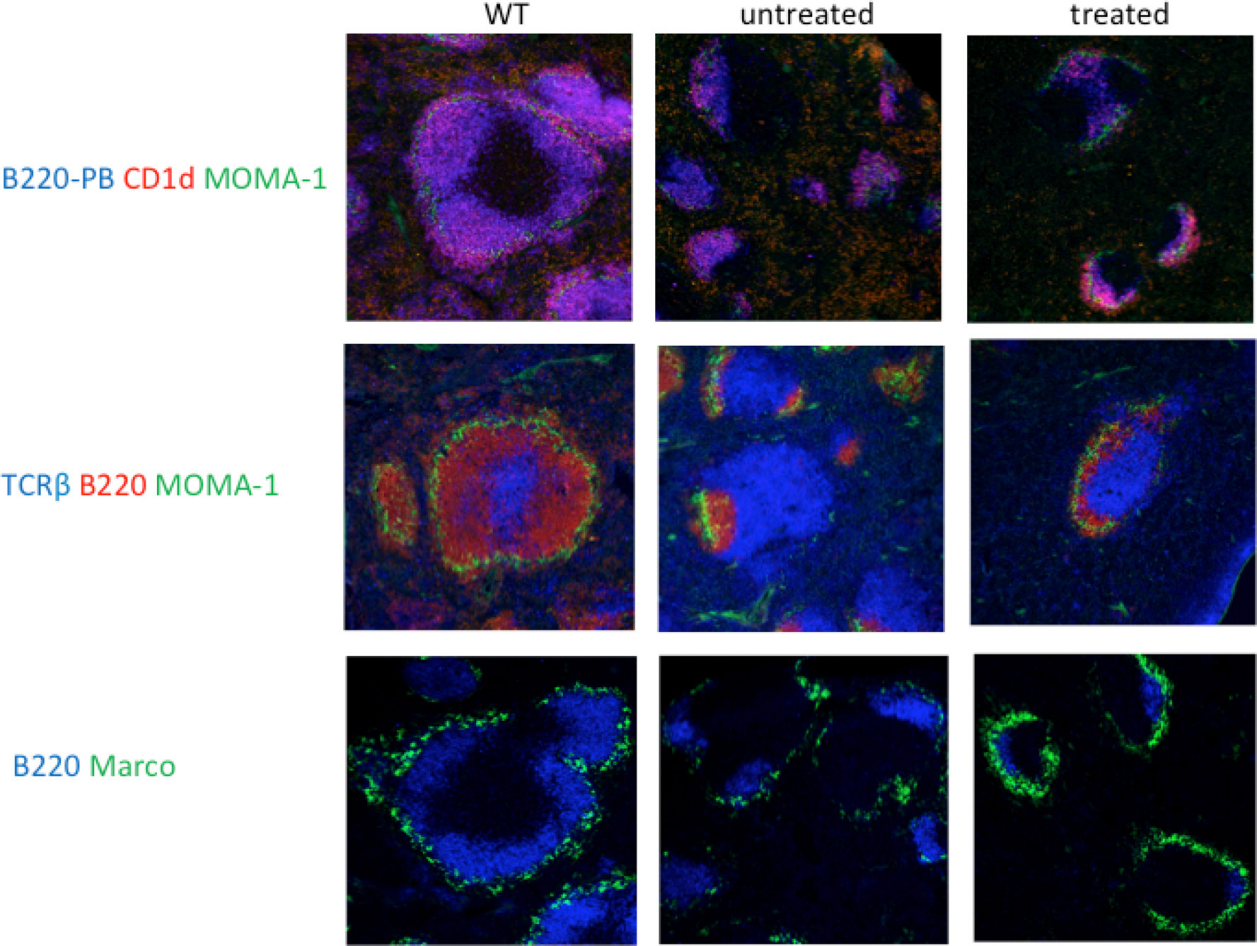
Characterization of the spleen architecture following treatment with Pip6a-conjugated splice-switching PMOs. Cells were detected using specific monoclonal antibodies: B cells (B220), marginal zone B cells (B220/CD1d), T lymphocytes (TCRβ), metallophilic macrophages (MOMA-1) and marginal zone macrophages (MARCO).

To investigate the effect of the treatment on the follicular structure of the spleen we performed immunohistochemistry analysis (Fig. 6). We stained for B cells (B220), MZ B cells (B220/CD1d), metallophilic macrophages (MOMA-1), T lymphocytes (TCRβ) and MZ macrophages (MARCO). The data show that there is a normalization of the splenic follicular structure with a more organized follicle where the specialized macrophages of the marginal zone surrounded the B cell areas and with the marginal zone B cells being localized to the correct position within the marginal zone and next to the metallophilic macrophages.

More specifically we observed that the white pulp in *BTK*/*Tec* double-deficient mice was altered and only a few clearly defined, but small follicular structures were observed. The majority of the B220^+^ B cell areas appeared to be loosely associated with T cell areas and no clear and distinct surrounding anti–MOMA-1 staining was observed, indicating an increased discontinuity of the marginal zone compared with *BTK*/*Tec* null mice. Germinal centers were also detected in *BTK*/*Tec* double-deficient mice. Interestingly, a relative increase in CD21^+^ CD23^lo^ MZ B cells was observed in *BTK*/*Tec* double-deficient mice when compared to WT, *Tec* ko or *BTK* ko animals.^27^

**Fig. 7:**
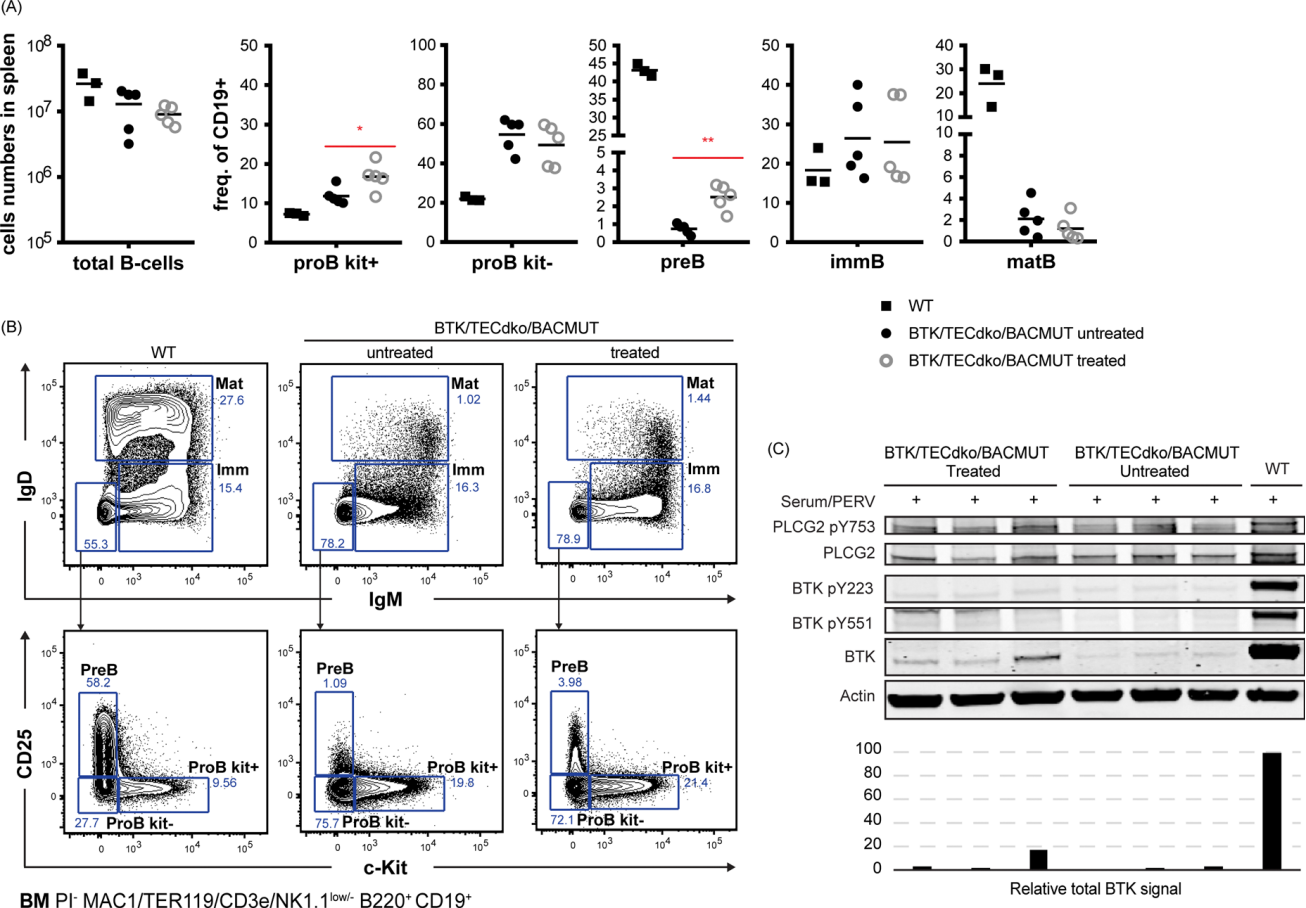
Characterization of the bone marrow B cell compartment following treatment with Pip6a-conjugated splice-switching PMOs. Panel (A), total (B) cells and B-lineage subpopulations, c-kit^+^ pro-B cells, c-kit^−^ pro-B cells, pre-B cells, immature B cells and mature B cells. Panel B, Gating strategy for identification of indicated B-cell subsets. Prior gating was performed as indicated below the fluorescence-activated cell sorting plots. Propidium iodide (PI) was used for dead cell discrimination. Panel (C), western blot showing expression of total *BTK*, PLCG2 and actin (loading control) as well as activated forms as detected by phosphorylation-specific antibodies for *BTK* pY223 and pY551 and PLCG2 pY753 in individual mice. The total *BTK* levels are presented in the bar graph.

We also studied the B cell compartment in the bone marrow (Fig. 7). As can be seen, both the frequency of ProB cKit^+^ and pre-B increased in treated animals, and this alteration is statistically significant (Panel A).

Our results demonstrate that different chemistries can be used for splice-switching in B lymphocytes. A major obstacle in the antisense field is the targeted delivery, especially to extrahepatic sites. Numerous modifications have been tested involving the sugar, nucleobase and internucleotide linkage to improve stability, cellular uptake and *in vivo* distribution.^7,28^ The most promising chemical modifications, phosphorothioate (PS) and phosphorodiamidate morpholino oligomer (PMO), as well as the 2′-substitution on the ribose sugar, have been tested in several clinical studies and approved as drugs by FDA.^29^ Our previous study showed for the first time the successful correction of an intronic mutation in the *BTK* gene by steric blocking of a cryptic splice-site both *in vitro* and *in vivo*, achieving efficient splice-switching into hard-to-transfect primary B cells.^8^ However, the functional impact of this correction has never been assessed *in vivo*.

In this current study, we decided to screen several new chemistries *in vitro* and, moreover, to select the best compounds and evaluate their efficacy using the BAC-transgenic model. Since humoral immunity in *BTK*-deficient mice is much less impaired as compared to humans with XLA, we now bred the mice onto a *BTK*/*Tec* double knock-out background, which resembles human XLA in terms of the humoral deficiency.^15^

We first selected the most efficient SSOs from our previous study (SSO 186 and SSO 187) targeting predicted ESE and 5′-cryptic splice-site proximal to pseudo-exon respectively. We subsequently compared the efficacy of SSOs made from LNA, tcDNA and the novel phosphoryl guanidine (PGO) chemistry. The neutral chemistries were thereafter combined with cell-penetrating peptide Pip6a and compared to the B-PMO conjugates that we studied previously.^8^

To achieve efficient targeting in our double knock-out mouse model we first tested CPP-conjugated SSOs in our *in vitro* reporter model. We here used CPP-PMO conjugates. PMO, an uncharged nucleic acid with a morpholino group and phosphorodiamidate linkages,^30^ has been approved by the FDA for the treatment of Duchenne's muscular dystrophy, *e.g. via* exon 51 skipping.^2^ In a number of studies, the bioavailability of PMOs has been improved by direct conjugation to CPPs and such peptide-PMOs have been successfully exploited in preclinical studies of DMD by using exon skipping strategy for dystrophin rescue.^31–33^ Especially the optimized CPP platform named Pip-peptides with a designed hydrophobic core has been shown to be potent in dystrophic mouse models and reach hard to target sites such as cardiac muscle and CNS.^25,34,35^ We compared our previously tested B-PMO conjugate to the more efficient variant Pip6a-PMO.^20,21^ Strikingly, Pip6a conjugated SSO 186 PMO demonstrated two-to-three-fold more activity in a concentration range of 0.625–5 μM which is in line with the previous reports.^36^ Pip6a is a highly cationic charged peptide with a hydrophobic core, which has been shown, in conjugation with charged neutral oligonucleotides (PMO, PNA), to be efficiently taken up by cells.^37^ This property likely contributes to the enhanced efficacy observed when Pip6a is conjugated with SSOs, as seen in our comparative analyses with B-PMO conjugates. This prompted us to test the novel phosphoryl guanidine (PGO) modification in conjugation with Pip6a. Even though unconjugated PGO SSOs 186 and 187 showed very minor activity *in vitro*, Pip6a conjugation enhanced their efficacy to a similar level as with Pip6a-186 PMO. In order to evaluate the efficacy of Pip6a conjugated to PMO or PGO SSOs, we compared the efficiency of splice-switching in primary B cells derived from our previously described human BAC transgenic mouse model with a full-length mutated human *BTK* pre-mRNA. Interestingly, evaluation of *BTK* protein levels after 48 h showed similar efficiency for SSOs Pip6a PMO 186 and Pip6a-PGO-186, whereas B-PMO 186 and Pip6a-PGO-187 had minor restoration. While the PGO-modification has been previously reported to efficiently restore the sensitivity to vinblastine in tumor cells by targeting *MDR1* RNA,^18^ this is the first time that their efficiency has been demonstrated in a primary B cell setting. Although LNA and tcDNA SSOs showed high activity in a reporter cell line setting, we have chosen CPP-conjugates of the neutral chemistries for *in vivo* testing owing to the nature of hard to target B cells. Further studies are needed to investigate LNA and tcDNA modified SSOs in an *in vivo* setting for targeting B cells.

Next, we tested the Pip6a PMO conjugate in the aforementioned double knock-out mouse model in order to study the functional activity of restored *BTK* protein after systemic administration. We used the Pip6a 186 PMO owing to its efficiency as well as having enough material for a long-term repeated dosing study. Initially, we tested the optimal dose for *BTK* restoration in the BAC transgenic mouse model. Intravenous injection of Pip6a 186 PMO revealed efficient *BTK* restoration at doses of 4 mg kg^−1^ and 12 mg kg^−1^ both in the spleen and bone-marrow cells isolated after 48 h. In order to monitor relevant functional change, we injected the *BTK*/*Tec* double-deficient mice with 10 mg kg^−1^ Pip6a-PMO 186 2 times a week during 3 weeks and investigated restoration of the B cell lineage by FACS and immunohistochemistry of the bone marrow and spleen. Analysis of the spleen compartment revealed that MZ B cells were normalized in comparison to non-treated animals. Moreover, we observed a tendency for an increase in the T1 B-cell subset, whereas follicular mature B cells did not show any augmentation. We further analyzed the *BTK* expression and the phosphorylation status upon activation as an indicator for a functional protein, as outlined before,^8^ and found clear signs of activation upon stimulation suggesting that the molecule is intact. To this end, phosphorylation of *BTK* at Y551 enhances its catalytic activity and results in its autophosphorylation at position Y223, although Y223 phosphorylation is functionally dispensable.^38^ Moreover, a fully active *BTK* phosphorylates PLCγ2 at position Y753. Although we could detect *BTK* in both splenic and bone marrow B cells, only the treated animal with the highest *BTK* expression in splenic B cells showed significant Y551 and Y223 phosphorylation. This suggests that treatment more often than intravenous infusions 2 times a week during 3 weeks is necessary. In our previous study using a B-PMO construct, treatment 3 times per week yielded higher expression.^8^

We also investigated the spleen architecture by immunohistochemistry in order to see the early stage changes in the lymphoid follicle. Staining of the spleen tissue sections for B cells (B220), Marginal zone B cells (B220/CD1d), metallophilic macrophages (MOMA-1) and Marginal zone macrophages (MARCO) revealed a normalization in the splenic follicular compartment. Moreover, we have also checked for the B cells in the bone marrow compartment. FACS analysis showed that treated animals have increased ProB cKit^+^ and pre-B cell numbers indicating developing B cells.

To this end, earlier studies have utilized lentiviral-based gene delivery into hematopoietic stem cells (HSCs) following transplantation in *BTK*/*Tec* double knockout mice, leading to significant restoration of major splenic B cell subsets and bone marrow B cell populations.^39,40^ In contrast, our non-viral approach partially restored the B cell phenotype, primarily normalizing splenic MZ B cells and increasing the proportion of ProB cKit^+^ and pre-B cells in the bone marrow. However, the transient SSO treatment seems to have been insufficient for driving all B cell subsets to full maturation. Targeting cells over a longer period could potentially lead to complete restoration of the B cell phenotype. Interestingly, while the phenotype was partly reverted in the *Btk*/*Tec* double-deficient mice, there were also changes that were unexpected. Thus, the increase in the transitional T1 subset differed from both normal and untreated mice and is likely caused by the instant loss of the developmental block, when *BTK* expression is turned on. It has been also shown that the differentiation into either follicular B cells (FoB) or marginal zone B cells (MZ B) is partially influenced by the strength of B-cell receptor (BCR) signaling.^41^ Notably, our ability to normalize only the MZ B cell population provides valuable insights into the BCR signaling threshold required for the maturation of distinct B cell subsets.

Hence, continued studies may give unique insights into the time course of B lineage subpopulation development during SSO-mediated phenotypic restoration. The SSO therapy here differs from the lentivirus-mediated treatment where all B cells develop from the HSC population. In contrast, the SSO therapy likely immediately affects all B lineage cells and not only the early progenitors derived from the HSCs. Thus, with SSOs, defined, developmental windows can be readily investigated by turning on and off the administration.

## Conclusions

This study provides evidence that SSOs made from a rather unique collection of different chemistries are able to correct aberrantly spliced *BTK* in B lymphocytes using a mouse model of X-linked agammaglobulinemia. To obtain sufficient restoration *in vivo* it was necessary to conjugate the ONs to cell-penetrating peptides. The effect was studied by a battery of different methods. Treatment with intravenous infusions 2 times a week during 3 weeks clearly reverted the phenotype, but longer treatment periods will be needed in order to restore the phenotype into normality. Owing to that the most mature B lineage cells, the plasma cells, do not need *BTK*, it may be possible, as a first step, to correct splicing in mature B lymphocytes, followed by immunization against infectious pathogens. Subsequently, the responding B cells would undergo maturation into plasma cells and, since human plasma cells can survive for decades, a transient treatment could have long-lasting clinical benefit. Hence our study provides the first evidence that repeated administration of SSO-CPP conjugates can lead to a partial restoration of B-cell development *in vivo*, demonstrating a functional phenotypic rescue and reinforcing the potential of SSO-based therapies for primary immunodeficiencies.

## Ethical statement

All animal work was performed according to the protocols approved by the Ethical Committee on Animal Experiments, Stockholm South, Sweden, S175-12, S7-11, 20091309-31/2. The animals were bred and maintained in accordance with Karolinska Institute's guidelines for animal welfare.

## Author contributions

The project was conceived by CIES and further developed by BB, YEH and RZ. The experiments were mainly carried out by BB and YHE with the help of QW, CH, DKM, DG, OPBW, TL, KEL, AB, MCIK, RM. SEA and MAW were involved in discussions related to the chemistry of oligonucleotides and FA, MJG, CL, DAS and JW synthesized and provided the oligonucleotides. BB and CIES wrote the manuscript and all authors were provided with the possibility to comment on the manuscript.

## Data availability

The data that support the findings of this study are available from the corresponding author, CIES, upon request. The relevant data supporting this article will be uploaded as part of the ESI.†

## Conflicts of interest

There are no conflicts to declare.

## Supplementary Material

CB-006-D4CB00312H-s001
