# Supplementary material for: Cell-penetrating peptide-conjugated, splice-switching oligonucleotides mitigate the phenotype in BTK/Tec double deficient X-linked agammaglobulinemia model
Source: RSC Chem Biol. 2025 Mar 31;6(5):761–71. doi: 10.1039/d4cb00312h (PMC11955834; doi:10.1039/d4cb00312h)
Supplement: CB-006-D4CB00312H-s001 [file CB-006-D4CB00312H-s001.zip › bestas_supplementary information_final2.docx]

**Supplementary Information**

**Cell-penetrating peptide-conjugated, splice-switching oligonucleotides mitigate the phenotype in Btk/Tec double deficient X-linked agammaglobulinemia model**

**Burcu Bestas*^a^, H. Yesid Estupiñán^a,m^ , Qing Wang^a^, Shabnam Kharazi^a^, Chenfei He^b^, Dara K. Mohammad^a^, Dhanu Gupta^a,f^, Oscar P. B. Wiklander^a,n^, Taavi Lehto^a,c,p^, Karin E. Lundin^a^, Anna Berglöf^a^, Mikael C. I. Karlsson^b^, Frank Abendroth^d,o^, Samir El Andaloussi^a,e^, Michael J. Gait^d^, Matthew J.A. Wood^f^, Christian J. Leumann^g^, Dmitry A. Stetsenko^i,j^, Robert Månsson^a,h^, Jesper Wengel^k^, Rula Zain^a,e,l^, C. I. Edvard Smith^a,e^**

ᵃ Department of Laboratory Medicine, Karolinska Institutet, ANA Futura, Alfred Nobels Allé 8 Floor 8, SE-141 52 Huddinge, Sweden, [edvard.smith@ki.se](mailto:edvard.smith@ki.se)
ᵇ Department of Microbiology, Tumor and Cell Biology, Karolinska Institutet, Stockholm, Sweden

^c^ Institute of Techology, University of Tartu, Tartu, Estonia
ᵈ Medical Research Council Laboratory of Molecular Biology, Cambridge CB2 0QH, UK
ᵉ Karolinska ATMP Center, Karolinska Institutet, Karolinska University Hospital, SE-171 76 Stockholm, Sweden
^f^ Department of Paediatrics, University of Oxford, Oxford OX3 7TY, UK
^g^ Department of Chemistry and Biochemistry, University of Bern, Freiestrasse 3, CH-3012 Bern, Switzerland
^h^ Department of Clinical Immunology and Transfusion Medicine, Karolinska University Hospital, Stockholm, Sweden
^i^ Institute of Cytology and Genetics, Siberian Branch of the Russian Academy of Sciences, 10 Lavrentiev Ave., Novosibirsk 630090, Russia
^j^ Department of Physics, Novosibirsk State University, 2 Pirogov Str., Novosibirsk 630090, Russia
^k^ Department of Physics, Chemistry and Pharmacy, Biomolecular Nanoscale Engineering Center, University of Southern Denmark, Odense, Denmark
^l^ Centre for Rare Diseases, Department of Clinical Genetics and Genomics, Karolinska University Hospital, SE-171 76 Stockholm, Sweden
^m^ Departamento de Ciencias Básicas, Universidad Industrial de Santander, Bucaramanga, Colombia
^n^ Breast Center, Karolinska Comprehensive Cancer Center, Karolinska University Hospital, Stockholm, Sweden
^o^ Department of Chemistry, University of Marburg, Marburg, D-35043, Germany

^p^ Institute of Pharmacy, Faculty of Medicine, University of Tartu, Nooruse 1, 50411 Tartu, Estonia

Corresponding author: [edvard.smith@ki.se](mailto:edvard.smith@ki.se)

**Materials**

SSOs

A list of the SSOs used is presented in Supplementary Table 1. SSOs used in this study were designed as described in our previous publication (8). 2’-O-Methyl RNA oligonucleotides containing phosphoryl guanidine (PGO) groups were assembled by a modified protocol of automated solid-phase β-cyanoethyl phosphoramidite synthesis, where Staudinger reaction with 2-azido-1,3-dimethylimidazolinium hexafluorophosphate was substituted for iodine oxidation (17,18). LNA-containing ONs were synthesized at the University of Southern Denmark. PMO-based ONs were purchased from Gene Tools and conjugation to B-PMO and Pip6a made at the MRC Laboratory of Molecular Biology. Tricyclo-DNA (tcDNA) SSOs were from Department of Chemistry and Biochemistry, University of Bern. Supplementary Figure 1 displays solid-phase synthesis of 3′-aminohexyl 2′-OMe PGO and subsequent peptide conjugation, and Supplementary Figure 2 illustrates synthesis of peptide conjugated phosphorodiamidate morpholino oligomers (P-PMO). Supplementary Figure 3 presents the structures of the Pip6a and B peptides.

Supplementary Table 1

| ssON | Sequence, 5′-3′ |
| --- | --- |
| tcDNA 186 | GAGTTCTCAGGATGT |
| tcDNA 187 | AGACTTACCACTTCC |
| LNA 186 | g*A*guucu*C*aggau*G*u |
| LNA 187 | a*G*ac*T*ua*C*ca*C*uu*C*c |
| PG-186 | c^*^a^*^g^*^a^*^g^*^u^*^u^*^c^*^u^*^c^*^a^*^g^*^g^*^a^*^u^*^g^*^u^*^a^*^ |
| PG-187 | g^*^a^*^g^*^a^*^c^*^u^*^u^*^a^*^c^*^c^*^a^*^c^*^u^*^u^*^c^*^c^*^u^*^u^*^ |
| Pip6a-PMO 186 | 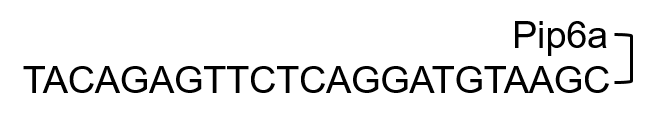 |
| B-PMO 186 | 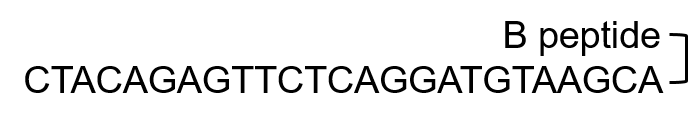 |
| Pip6a-control (DMD) | 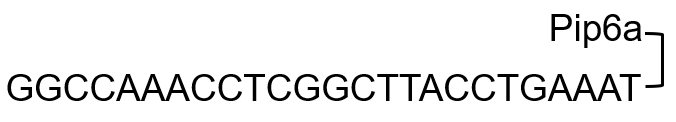 |
| Pip6a-PG-186 | 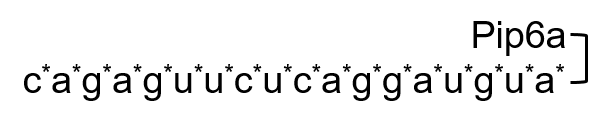 |
| Pip6a-PG-187 | 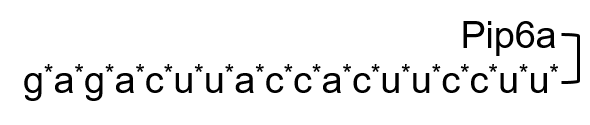 |
| PMO-BTK | CTACAGAGTTCTCAGGATGTAAGCA |

Notes: tcDNA nucleotides (underlined uppercase) had a phosphodiester (PO) backbone (16). LNA 186 and 187 contain 2′-*O*-methyl RNA bases (in lowercase) with a phosphorothioate (PS) backbone, while LNA bases are represented in uppercase italics. PMOs are in uppercase. PGO-186 and PGO-187 have 2′-*O*-methyl backbone and all phosphates are replaced with phosphoryl guanidine Dmi (1,3-dimethylimidazolidine-2-imine) groups >P(=O)–N=C(NMeCH_2_)_2_ (17,18). Both oligos have 3’-aminohexyl linker 3’-O(CH_2_)_6_NH_3_^+^ except when conjugated to Pip6a peptide. * – phosphoryl guanidine groups (Dmi). B-peptide sequence: N-RXRRBRRXRRBRXB-C. Pip6a peptide sequence: N-RXRRBRRXR YQFLI RXRBRXRB (X denotes 6-aminohexanoic acid residues, B – beta-alanine residues).





**Supplementary Figure 1**. Solid-phase synthesis of 3′-aminohexyl 2′-OMe PGO on 3′-PT-Amino-Modifier C6 CPG and subsequent peptide conjugation to obtain peptide 2′-OMe PGO conjugates (P-PGO): (*i*) – solid-phase PGO synthesis; (*ii*) – conc. (~40%) aq. МеNH_2_ – conc. (~28%) aq. NH_3_ (1:1 v/v) (АМА), 65^о^С, 15 min; (*iii*) 100 mМ peptide, 300 mМ TSTU, 300 mМ DIEA, NMP, 0.5 h; (*iv*) 4 eq. activated peptide, NMP – DMSO, 37^о^С, 5 h. 1,3-Dimethylimidazolidine-2-imine (Dmi) group is shown in red. DMTr – 4,4’-dimethoxytrityl, DIEA – *N*,*N*-diisopropylethylamine, NMP – N-methylpyrrolidin-2-one, TSTU – 1-succinimidyloxy-*N*,*N*,*N'*,*N'*-tetramethyluronium tetrafluoroborate.





**Supplementary Figure 2**. Synthesis of peptide-conjugated phosphordiamidate morpholino oligomers (P-PMOs): (*i*) – activation of peptide acid: 100 mМ peptide, 200 mМ HBTU – HOBt, 800 mМ DIEA, NMP, 3 min; (*ii*) 2.5 eq. activated peptide, NMP – DMSO, 37^°^С, 5 h; DIEA – *N*,*N*-diisopropylethylamine, NMP – *N*-methylpyrrolidin-2-one, HBTU – 1*H*-benzotriazol-1-yloxy(dimethylamino)methylidene]-dimethylazanium hexafluorophosphate, HOBt – 1-hydroxy-1*H*-benzotriazole.

**Supplementary Figure 3.** Structures of the Pip6a peptide and the B peptide in the conjugates.

**Methods**

**Cell culture and transfections**

The U2OS cell line stably expressing pEGFPLuc/Btkint4mut was generated as described before (8). For SSO transfections, cells were plated to reach 70% confluency at the day of transfection in a 24-well plate. The cells were incubated further for 3 days with SSOs in DMEM containing 10% FBS.

**Luciferase assay**

For analysis of luciferase activity in the stably transfected U2OS cell line, cells were lysed in 300 μl RLT buffer (Promega), and 20 μl lysate was used for measuring luciferase activity with the Luciferase Assay Kit (Promega). Luminescence was detected using a FluoStar OPTIMA (BMG Labtech). Total protein was detected using the MicroBCA Kit (Pierce) and used for normalization of luminescence.

**Semiquantitative reverse transcription (RT-PCR)**

Cells stably transfected with the reporter gene (U2OS/EGFPLucBTKint4mut) were washed twice with 1xPBS, and RNA was directly isolated from the plated cells using the RNeasy Plus Kit (Qiagen).

RNA was reverse transcribed into cDNA using the First Strand cDNA Synthesis Kit for RT-PCR (AMV-Roche). PCR was performed with HotStarTaq Plus DNA polymerase (Qiagen). An amount of 100 ng of total RNA was used. Primers for determining splice correction in the U2OS/EGFPLucBTKint4mut cells were as follows: EGFPLuc Fwd-5′-ctggtgccaaccctattctccttc; EGFPLuc Rev-5′-ccagatccacaaccttcgcttcaa-3′. The primers used in the case of human transgene, primary mouse B cells were as follows: BTK Fwd, 5′-cacacaggtgaactccagaaag, and BTK Rev, 5′-agagatactgcccatcgatccaga-3′. Cycling conditions were as follows: 95 °C for 5 minutes, 30 cycles of 94 °C for 30 seconds, 57 °C for 30 seconds, 72 °C for 30 seconds, and finally 72 °C for 10 minutes. As an RNA quality control 18S ribosomal RNA (18s rRNA) was also analyzed by PCR. The primer sequences were as follows: 18S Fwd-5′ gtaacccgttgaaccccatt; 18S Rev-5′-ccatccaatcggtagtagcg. Cycling conditions were as follows: 95°C for 5 minutes, 12 cycles of 94 °C for 30 seconds, 57°C for 30 seconds, 72 °C for 30 seconds, and finally 72 °C for 10 minutes. Samples were run on Fragment Analyzer (Agilent). Corrected peak area was used to quantify the WT mRNA.

**Mice and injections**

BAC transgenic mice were bred on BTK/TEC dKO background (C57BL/6). BtkTec^-/-^  mice was kindly provided by Wilfried Ellmeier (15) and were bred and maintained in the pathogen-free barrier facilities at Karolinska Institute. BAC transgenic mice were genotyped as described in our previous paper (8). For the dKO BAC transgenic animals, ear or tail biopsies were collected and DNA was isolated (DNeasy Blood & Tissue Kit, Qiagen). PCR with HotStarTaq Plus DNA polymerase (Qiagen) was performed. Primers were designed to amplify *Tec* and *Btk genes*. Genotyping was conducted by amplifying human *BTK* (exons 1, 11, and 19) using previously described protocol (8). Additionally, amplification of mouse *Btk* and *Tec* was performed to verify the absence of both genes and confirm the presence of human *BTK* gene*.* Samples were run on 1.25% agarose gel and analyzed by Gel Doc system (Bio-Rad).

5 BACMUT Btk/Tec^-/-^ mice were injected intravenously 2 times a week during 3 weeks with 10 mg/kg Pip6a-PMO 186 . Five untreated BACMUT Btk/Tec^-/-^ and 3 WTs as served as controls.

All animal work was performed according to the protocols approved by the Ethical Committee on Animal Experiments, Stockholm South, Sweden, S175-12, S7-11, 20091309-31/2. The animals were bred and maintained in accordance with Karolinska Institutet’s guidelines for animal welfare.

**Mouse B lymphocyte preparation, culturing, and transfections**

Purified splenic mouse B lymphocytes were obtained using an EasySep Mouse B Cell Enrichment Kit, which enriches cells through negative selection (StemCell Technologies). The purity of isolated B cells was checked by staining of the cell population with a PE-anti-mouse CD19 (BD Pharmingen), as analyzed by flow cytometry. Purity levels were always ≥90%.

After isolation, B cells were cultured in Iscove’s Modified Dulbecco’s Medium (IMDM) containing 15% FBS and 50 μM β-mercaptoethanol (Gibco, Life Technologies), with addition of 5 μg/ml CpG ON (obtained from DNA Technology A/S) for stimulation and improved survival of the cells in culture (1). Lymphocytes were thus incubated with CpG ON for 3 to 4 hours before proceeding with the transfections.

For the gymnosis/naked uptake experiments, cells were plated at 1.5 × 10^6^ cells per well on the day of transfection. The same CpG treatment was done as described above. The SSOs were given directly to the cells. B-PMOs were incubated at 37 °C for 30 minutes and sonicated for a few seconds in a water bath before use. The cells were incubated further for 3 to 4 days with ssONs.

**Flow cytometry**

Dissected spleens and BM were crushed in phosphate-buffered saline (PBS) with 2% fetal calf serum (FCS) and filtered through a 70-mm nylon filter. Cells from the peritoneal cavity were centrifuged. All cells were then resuspended in PBS with 2% FCS, subsequently Fc-blocked using anti-CD16/32 (93; BD Biosciences) and stained with fluorochrome-conjugated antibodies (Abs) as previously described (2).

For Ab panels used, see Supplementary Table 1. Propidium iodide was used to discriminate dead cells. Peripheral B-staining was used for spleens and peritoneal cavity, B-cell progenitor panel was used for BM cells. Data were acquired on a FACSAria IIu (BD Biosciences) and analyzed using Flowjo 9.9.6.

**Western blot**

Cell lysates were obtained, after washing cells in PBS twice, by incubation with lysis buffer (50 mM HEPES, pH 7.0, 120 mM NaCl, 10% glycerol, 1% NP-40, 0.5% sodium deoxycholate) supplemented with protease inhibitors (Complete Mini, Roche) for 30 minutes with repeated vortexing. Finally, the lysates were cleared by centrifugation. Proteins were separated on gradient 4%–12% SDS Bis-Tris NuPAGE gels (Life Technologies) and transferred onto nitrocellulose membranes using the Iblot system (Invitrogen). The membranes were then blocked with LI-COR Blocking Buffer (LI-COR Biosciences GmbH) and probed with specific primary antibodies. Western blots signals were scanned by using Odyssey Imager from LI-COR Biosciences GmbH. The following primary antibodies were used for detection: actin (A5441, Sigma-Aldrich), BTK (270-284, Sigma-Aldrich), phospho-BTK (pY551) (558129, BD), and BTK (611117, BD). The following secondary antibodies were used for detection: goat anti-mouse 800CW, goat anti-rabbit 800CW, goat anti-mouse 680LT, or goat anti-rabbit 680 (all from LI-COR Biosciences GmbH). Actin was used as loading control. Quantitative analysis of BTK protein was calculated as the percentage of relative intensity by ImageJ software according to the manufacturer’s protocol. Phosphorylation was analyzed following a 4-hour serum starvation period, after which cells were activated with serum [FBS (2%)] and pervanadate [0.02% H2O2, 1.6% Tyrode’s salt solution (Sigma-Aldrich) and 0.22 mM Na3VO4 (Sigma-Aldrich)] and for 5 minutes at room temperature.

**Immunohistochemistry**

Spleens were frozen in cryostat embedding medium (Caltag NO. 05-9801; Bio-optica) and 8-mm-thick sections were cut using a cryostat microtome. After overnight drying, the slides were fixed in acetone. Before staining, slides were blocked with 5% goat serum (DakoCytomation) in PBS, and then were stained with the fluorescent conjugated Abs and biotin Ab in the 4 ℃ overnight. Afterwards, the sections were washed and subsequently incubated with the fluorescent conjugated streptavidin for 1 h at room temperature. The reagents used for immunohistochemistry were biotin-conjugated anti-B220 (clone RA3-6B2; Caltag NO. 103204; Biolegend), PE-conjugated anti mouse CD1d (clone 1B1; Caltag NO. 123510; Biolegend), FITC-conjugated anti mouse CD169 (clone MOMA-1; Caltag NO. MCA947F; Bio-Rad-antibodies), Alexa Fluor®647 streptavidin (Caltag NO. 405237; Biolegend), APC-conjugated anti-TCRβ (clone H57-597; Caltag NO.109218; Biolegend). The rat anti-MARCO IgG1mAb hybridoma ED31 was a generous gift from Prof. G. Kraal, and it was conjugated by Alexa Fluor™ 488 Antibody Labeling Kit (Catalog NO. A20181, Invitrogen). Slides were mounted with ProLong™ Diamond Antifade Mountant (Catalog NO. P36961, Invitrogen). Visual data were acquired with a confocal microscope (Zeiss LSM880), and recorded with the LSM Image software.

| Supplementary Table 2. Antibody panels used for cytometry. Unless otherwise indicated, fluorochromes defined in the detector column was utilized. BD biosciences, BD; BioLegend, BioL. | | |
| --- | --- | --- |
| **Detector** | **Peripheral B-cell staining** | **B-cell progenitors** |
| **APC** | CD93 ( AA4.1 ), BD | - |
| **APC-Cy7** | B220 (RA3-6B2) Alexa780, BD | B220 (RA3-6B2) Alexa780, BD |
| **BV421** | IgD (11-26c.2a), BD | cKit (2B8), BD |
| **BV605** | CD21 (7G6), BD | - |
| **BV711** | CD23 (B3B4 ), BD | - |
| **BV786** | - | IgD (11-26c.2a), BD |
| **PE** | - | CD25 (PC61.5), BD |
| **PE-Cy7** | IgM (11/41), BD | IgM (11/41), BioL |
| **PE-CF594** | CD19 (1D3), BD | CD19 (1D3), BD |


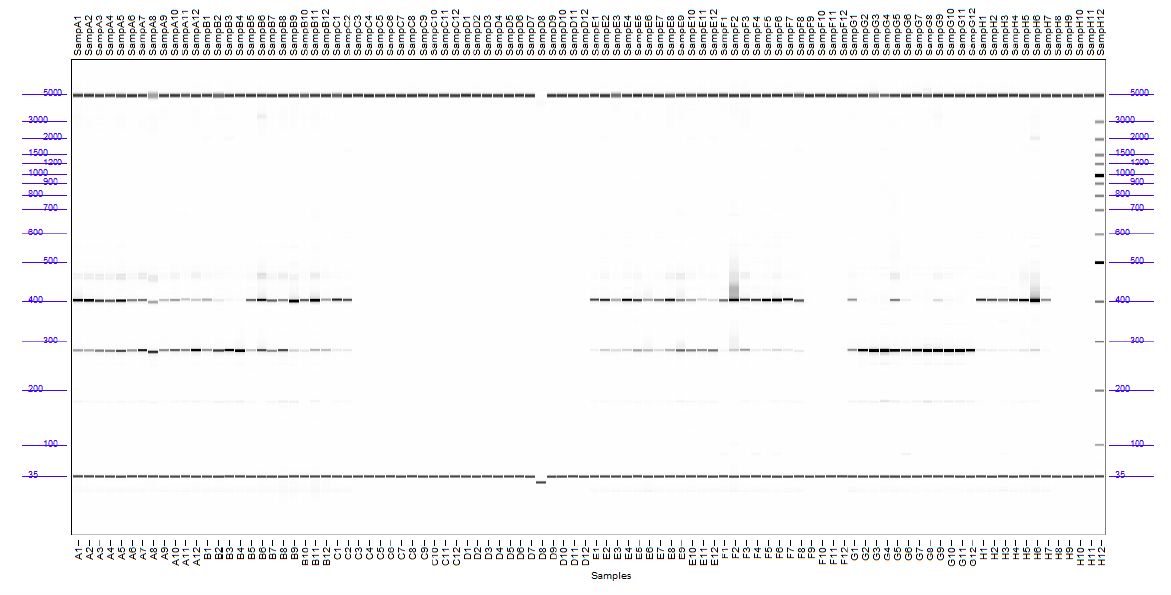


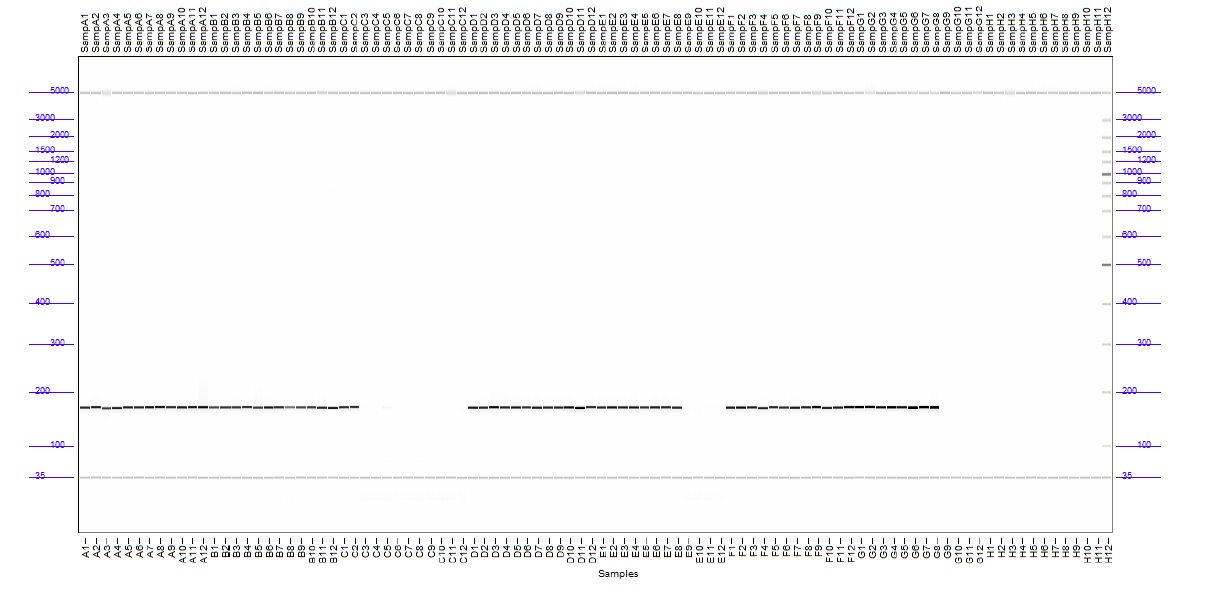


**Supplementary Figure 4.** Capillary electrophoresis image for Figure 1b. The upper panel displays mRNA bands for aberrant (mutant) and corrected forms (with *BTK* intron 4 excised). The lower panel shows the 18S ribosomal RNA band. Relevant lanes are highlighted with red rectangles. Lanes A5-A8: tcdna-187, A9-A12:tcDNA-186, B1-B4: LNA 186, B5-B8: LNA 187, B9-B12: PMO, C1-C2:UT. Size marker is shown in blue. 35 bp – 5,000 bp bands are the internal markers.


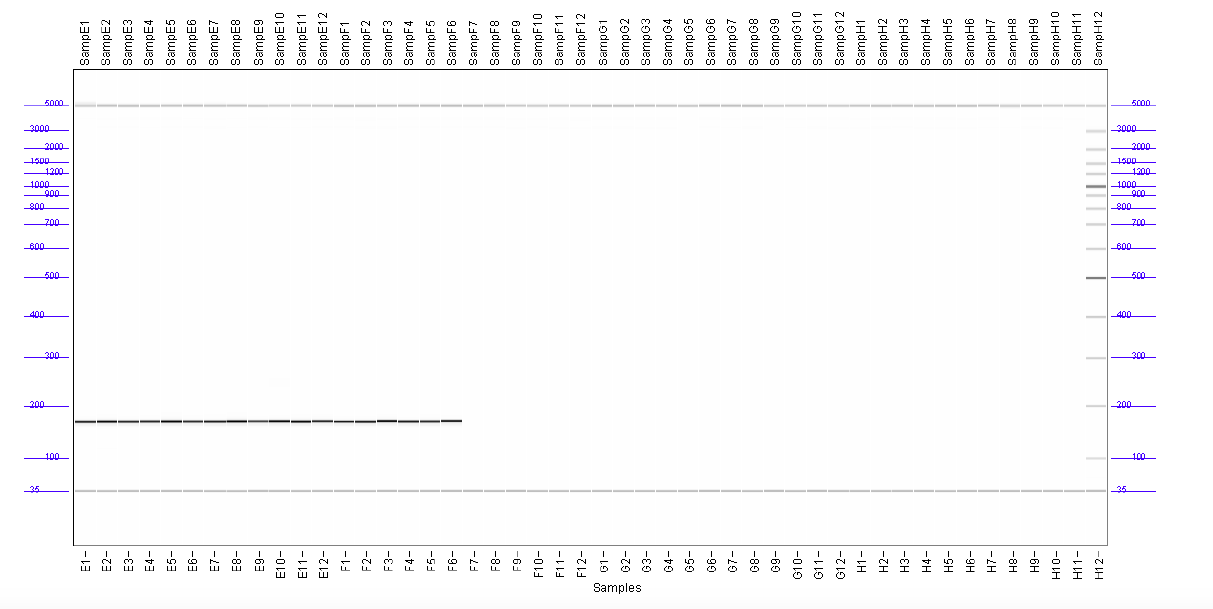


**Supplementary Figure 5**. Capillary electrophoresis image for Figure 2a. The upper panel displays mRNA bands for aberrant (mutant) and corrected forms (with *BTK* intron 4 excised). The lower panel shows the 18S ribosomal RNA band. Relevant lanes are highlighted with red rectangles. Upper panel lanes A1-A8: Pip6a-PMO 186, A9-B4: B-PMO 186, B5-B6:UT for *BTK* aberrant and WT mRNA variants. Lower panel lanes E1-F6 shows 18S ribosomal RNA band with the respective samples. Size marker is shown in blue. 35 bp – 5,000 bp bands are the internal markers.


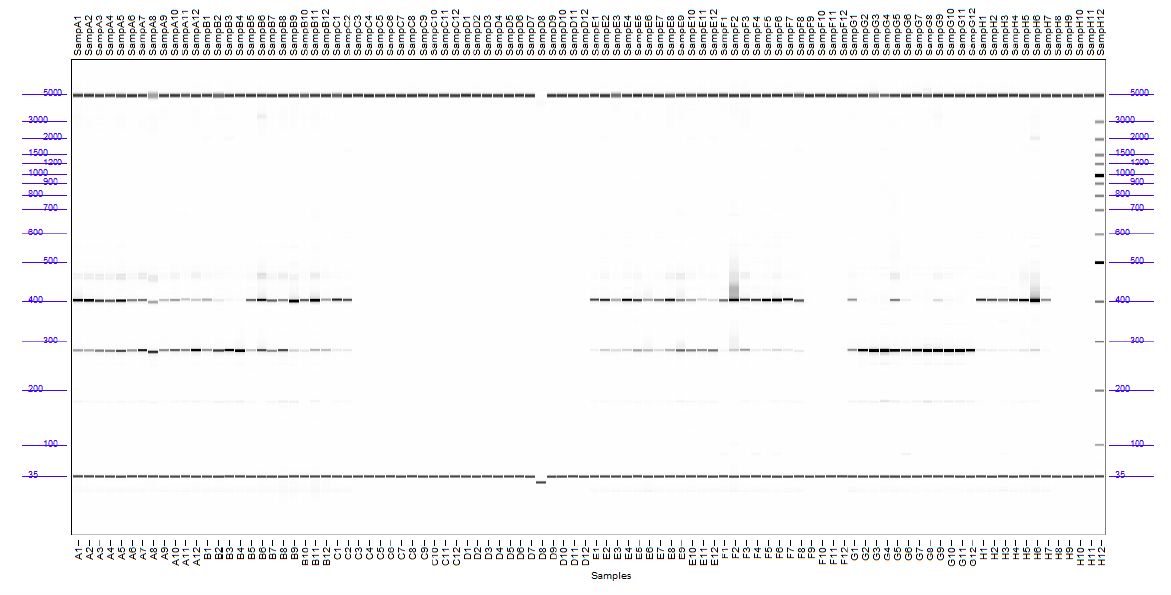


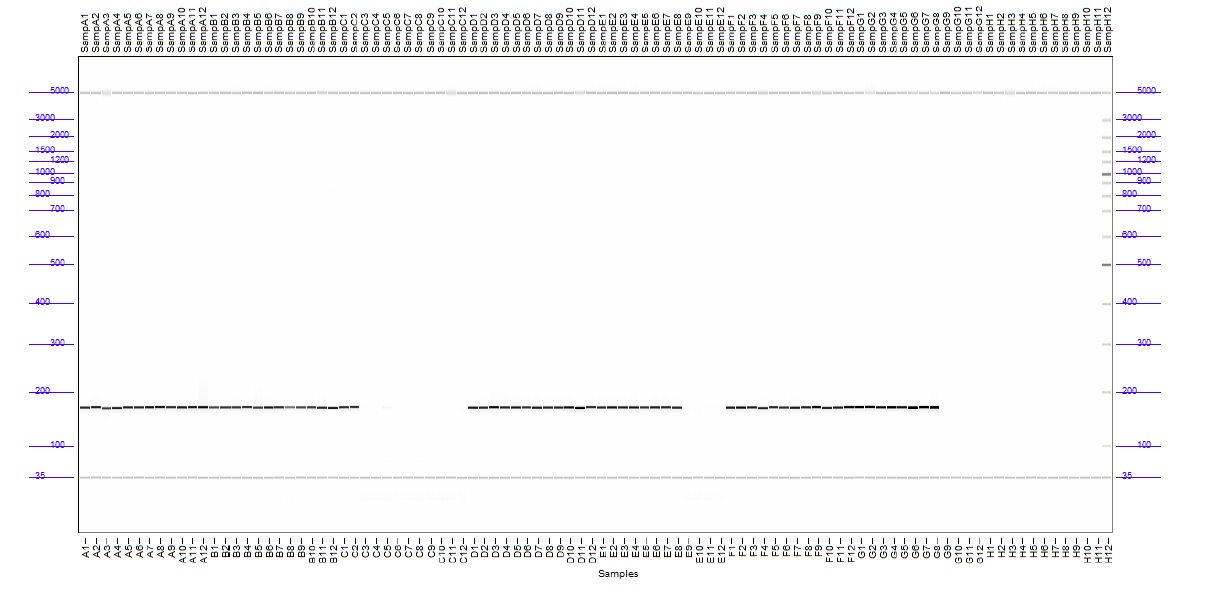


**Supplementary Figure 6.** Capillary electrophoresis image for Figure 2b. The upper panel displays mRNA bands for aberrant (mutant) and corrected forms (with *BTK* intron 4 excised). The lower panel shows the 18S ribosomal RNA band. Relevant lanes are highlighted with red rectangles. Upper panel lanes G1-G4: Pip6a-PMO 186, G5-G8: Pip6a-PGO-186, G9-G12: Pip6a-PGO-187, H1-H4 PMO-BTK 186, H5:UT for *BTK* aberrant and WT mRNA variants. Lower panel lanes F1-G5 shows 18S ribosomal RNA band with the respective samples. Size marker is shown in blue. 35 bp – 5,000 bp bands are the internal markers.


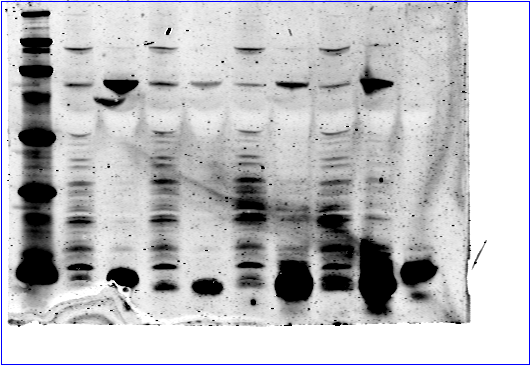

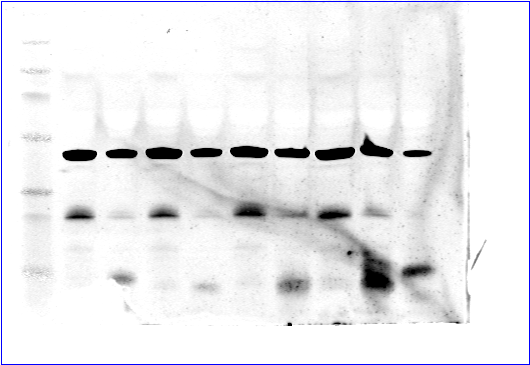

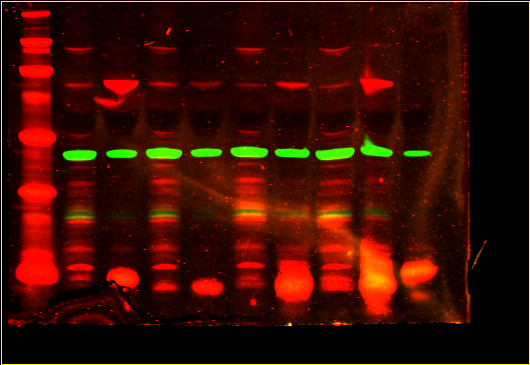


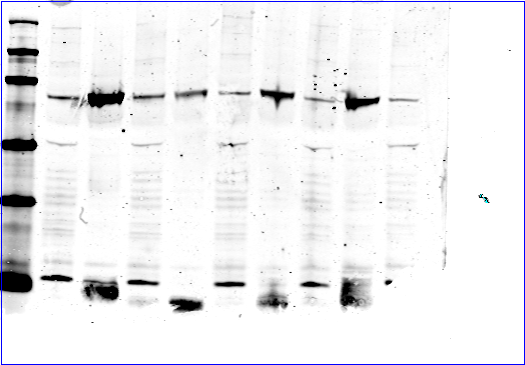

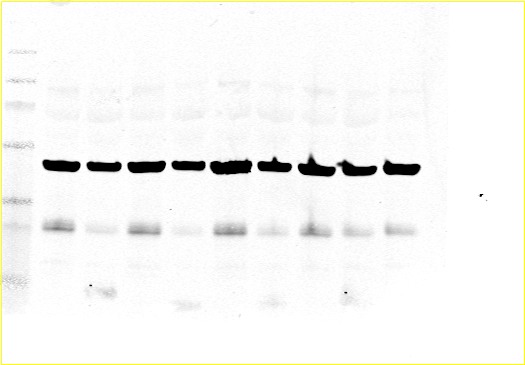

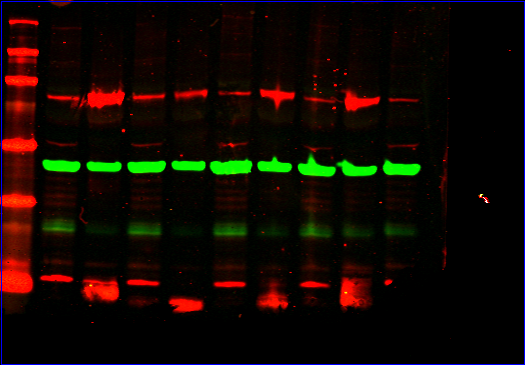


**Supplementary Figure 7.** Western blot image for Figure 3. Upper left panel shows the grayscale blot for BTK, while the upper middle panel displays the grayscale blot for Actin. Upper right panel combines both channels: BTK is represented in red (700 channel), and Actin in green (800 channel). The lower left and middle panels display the untreated control (indicated by a red box), cropped from these blots. In the main text image, a box has been added around the control sample to highlight it. Upper blots, Lane 1: Size Marker, lanes 2-3: Pip6a-PMO 186, lanes 4-5: B-PMO 186, lanes 6-7: Pip6a-PGO-187, lanes 8-9: Pip6a-PGO-186. The lower right panel shows a combination of both channels. Brightness and contrast adjustments were applied to all blots for clarity.


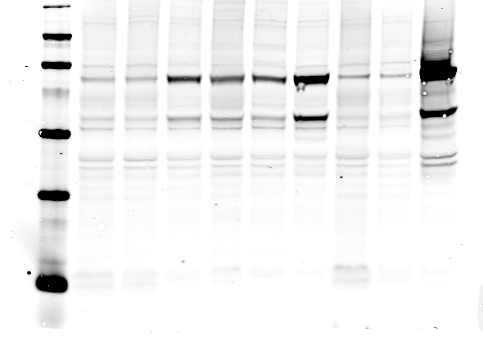

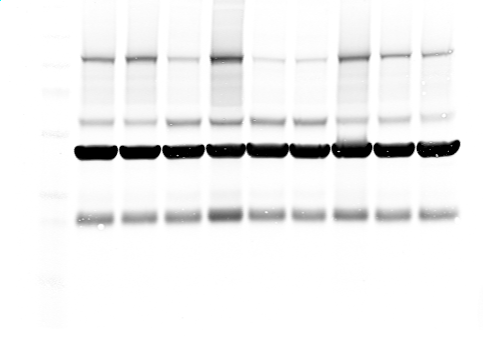

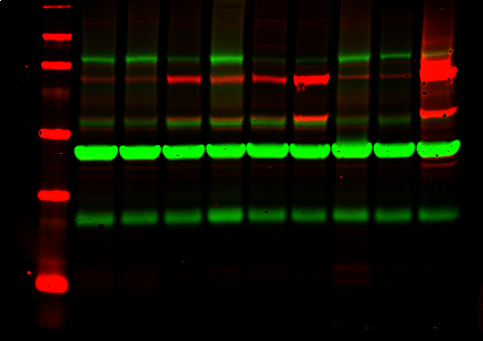


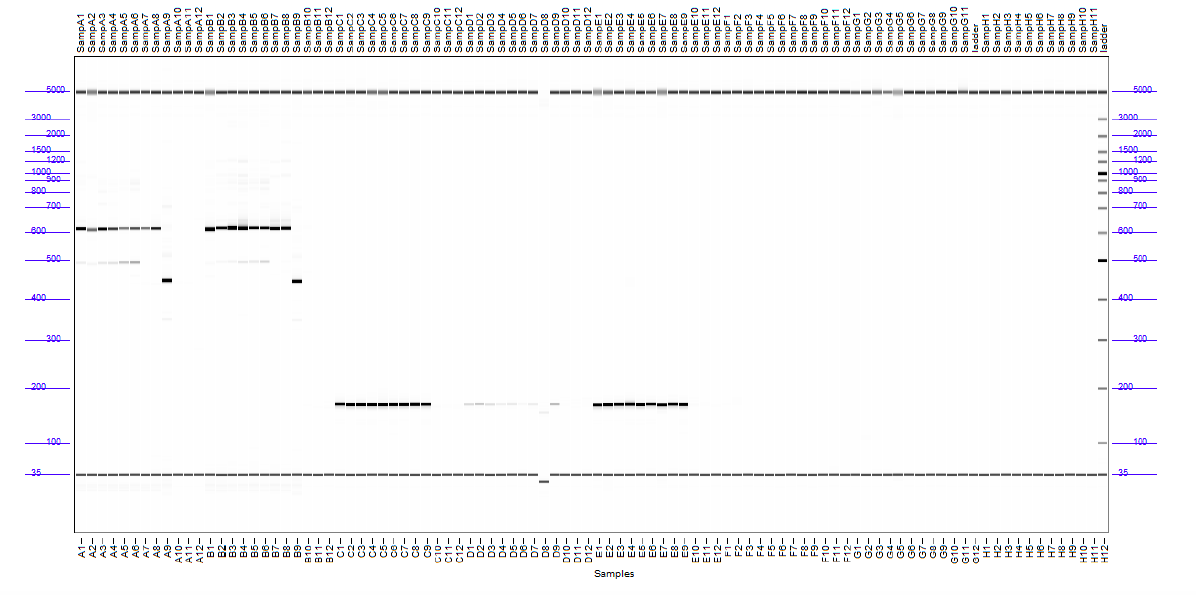


**Supplementary Figure 8.** Western blot and capillary electrophoresis images for Figure 4. Upper left panel shows the grayscale blot for BTK, while the upper middle panel displays the grayscale blot for Actin. Upper right panel combines both channels: BTK is represented in red (700 channel), and Actin in green (800 channel). Lane 1: Size Marker, lanes 2-3: 1mg/kg, lanes 4-5: 4mg/kg, lanes 6-7: 12 mg/kg, lanes 8-9: Control, lanes 10: WT. Brightness and contrast adjustments were applied to all blots for clarity.

The lower panel displays the capillary electrophoresis image. Lanes A1-A2 (1 mg/kg), A3-A4 (4 mg/kg), and A5-A6 (12 mg/kg) show spleen mRNA bands for both aberrant (mutant) and corrected forms (with BTK intron 4 excised), with A7-A8 serving as the control. Similarly, lanes B1-B2 (1 mg/kg), B3-B4 (4 mg/kg), and B5-B6 (12 mg/kg) display bone marrow mRNA bands for the same forms, with B7-B8 as the control. Lanes C1-C9 and E1-E9 represent the 18S ribosomal RNA bands for spleen and bone marrow, respectively.

A


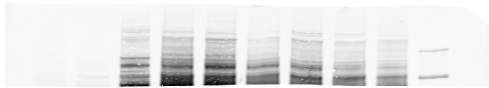


130 KDa

B


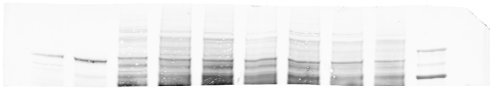


130 KDa

C


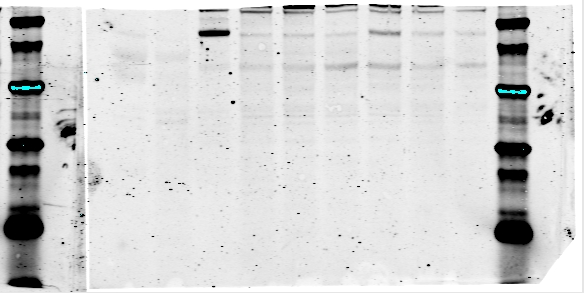


70 KDa

D


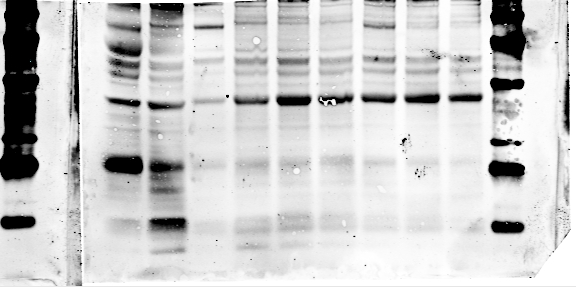


70 KDa

E


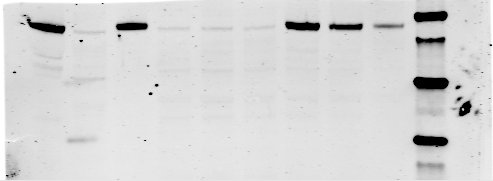


70 KDa

F


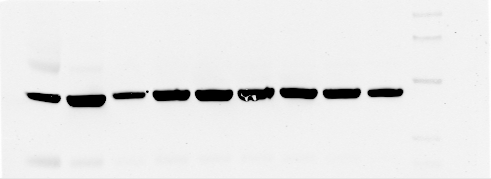


55 KDa

**Supplementary Figure 9**. Western blot images for Figure 5. Panel A: PLCG2 pY753, panel B: PLCG2, panel C: BTK pY223, panel D: BTK pY551, panel E: total BTK, panel F: actin (loading control) for spleen. During the acquisition pictures were flipped horizontally, visualizing the molecular weight indicator in the first well from left to right. All samples are placed as described order in the corresponding figure (2 – 4 wells Treated BTK/TECdko/BACMUT; 5 – 7 wells Unreated BTK/TECdko/BACMUT: 8 well WT). Brightness and contrast adjustments were applied to all blots for clarity.

A


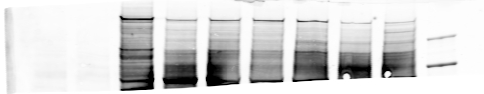


130 KDa

B


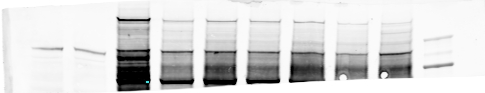


130 KDa

KDa

C


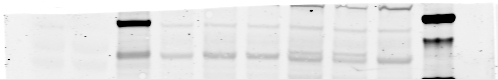


70 KDa

D


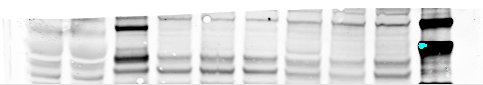


70 KDa

E


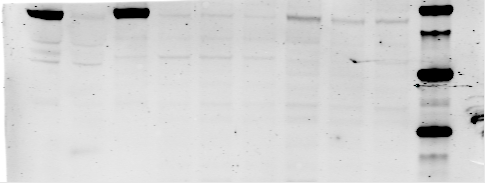


70 KDa

F


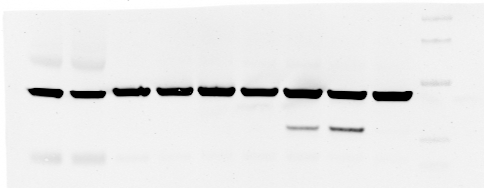


55 KDa

**Supplementary Figure 10.** Western blot images for Figure 7. Panel A: PLCG2 pY753, panel B: PLCG2, panel C: BTK pY223, panel D: BTK pY551, panel E: total BTK, panel F: actin (loading control) for bone marrow. During the acquisition pictures were flipped horizontally, visualizing the molecular weight indicator in the first well from left to right. All samples are placed as described order in the corresponding figure (2 – 4 wells Treated BTK/TECdko/BACMUT; 5 – 7 wells Unreated BTK/TECdko/BACMUT: 8 well WT). Brightness and contrast adjustments were applied to all blots for clarity.

**Supplementary Figure 11.** Analytical data of oligonucleotides conjugated to cell-penetrating peptides

**B-PMO 186**

Ac-RXRRBRRXRRBRXB-TACAGAGTTCTCAGGATGTAAGC

Yield: 19%

Chemical Formula: C_352_H_574_N_175_O_107_P_23_

Maldi-Tof MS: calc.[M+H^+^] 9691.89 found: 9692

HPLC:

**Pip6a-control**

Ac-RXRRBRRXRYQFLIRXRBRXRB-GGCCAAACCTCGGCTTACCTGAAAT

Yield: 35 %

Chemical Formula: C_425_H_692_N_199_O_124_P_25_

Maldi-Tof MS: calc.[M+H^+^] 11348.63 found: 11348

HPLC:

**Pip6a-PMO186**

Ac-RXRRBRRXRYQFLIRXRBRXRB-TACAGAGTTCTCAGGATGTAAGC

Yield: 17 %

Chemical Formula: C_406_H_659_N_190_O_116_P_23_

Maldi-Tof MS: calc.[M+H^+^] 10771.16 found: 10771

HPLC:

**Pip6a- PGO-186**

Ac-RXRRBRRXRYQFLIRXRBRXRB-c^*^a^*^g^*^a^*^g^*^u^*^u^*^c^*^u^*^c^*^a^*^g^*^g^*^a^*^u^*^g^*^u^*^a^*^

Yield: 46 %

Chemical Formula: C_416_H_653_N_177_O_134_P_18_

Maldi-Tof MS: calc.[M+H^+^] 10835.24 found: 10835

HPLC:

**Pip6a-PGO-187**

Ac-RXRRBRRXRYQFLIRXRBRXRB-g^*^a^*^g^*^a^*^c^*^u^*^u^*^a^*^c^*^c^*^a^*^c^*^u^*^u^*^c^*^c*u^*^u^*^

Yield: 68%

Chemical Formula: C_412_H_652_N_168_O_136_P_18_

Maldi-Tof MS: calc.[M+H^+^] 10693.14 found: 10693

HPLC:

**References**

1 M. Hasan, G. Lopez-Herrera, K. E. M. Blomberg, J. M. Lindvall, A. Berglöf, C. I. E. Smith and L. Vargas, *Immunology*, 2008, **123**, 239–249.

2 T. Bouderlique, L. Peña-Pérez, S. Kharazi, M. Hils, X. Li, A. Krstic, A. De Paepe, C. Schachtrup, C. Gustafsson, D. Holmberg, K. Schachtrup and R. Månsson, *Front Immunol*, 2019, **10**, 455.
